# Does Temporal Integration Occur for Unrecognizable Words in Visual Crowding?

**DOI:** 10.1371/journal.pone.0149355

**Published:** 2016-02-18

**Authors:** Jifan Zhou, Chia-Lin Lee, Kuei-An Li, Yung-Hsuan Tien, Su-Ling Yeh

**Affiliations:** 1 Department of Psychology, National Taiwan University, Taipei, Taiwan; 2 Department of Psychology and Behavioral Sciences, Zhejiang University, Hangzhou, China; 3 Graduate Institute of Brain and Mind Sciences, National Taiwan University, Taipei, Taiwan; 4 Neurobiology and Cognitive Neuroscience Center, National Taiwan University, Taipei, Taiwan; 5 Graduate Institute of Linguistics, National Taiwan University, Taipei, Taiwan; University of Leicester, UNITED KINGDOM

## Abstract

Visual crowding—the inability to see an object when it is surrounded by flankers in the periphery—does not block semantic activation: unrecognizable words due to visual crowding still generated robust semantic priming in subsequent lexical decision tasks. Based on the previous finding, the current study further explored whether unrecognizable crowded words can be temporally integrated into a phrase. By showing one word at a time, we presented Chinese four-word idioms with either a congruent or incongruent ending word in order to examine whether the three preceding crowded words can be temporally integrated to form a semantic context so as to affect the processing of the ending word. Results from both behavioral (Experiment 1) and Event-Related Potential (Experiment 2 and 3) measures showed congruency effect in only the non-crowded condition, which does not support the existence of unconscious multi-word integration. Aside from four-word idioms, we also found that two-word (modifier + adjective combination) integration—the simplest kind of temporal semantic integration—did not occur in visual crowding (Experiment 4). Our findings suggest that integration of temporally separated words might require conscious awareness, at least under the timing conditions tested in the current study.

## Introduction

Our conscious visual perception has a limit on recognizing objects that are closely clustered together, especially when they are located in the peripheral visual field. It has been shown that visual crowding impairs recognition of faces [1], letters [2] and numbers [3]. As such, visual crowding has been considered as a bottleneck for object perception [4,5].

Despite impaired conscious identification, some properties of crowded objects are still processed. For example, it has been demonstrated that processes of orientation [6,7], motion direction [8,9], and configuration [10,11] are preserved even under visual crowding. In addition, and probably most surprisingly, word meaning also survives crowding. Yeh, He, and Cavanagh [12] showed that unrecognizable crowded words led to semantic priming on the subsequently presented uncrowded target words. Later, Peng, Zhang, Chen, and Zhang [13] demonstrated an N400 priming effect on a crowded target word following a semantically related versus unrelated uncrowded prime word. These findings indicate that semantic information is activated even when a word is not consciously recognizable due to visual crowding.

Although these studies provided evidence for semantic access under visual crowding, they actually only measured responses to a target preceded by primes of single words. Thus, previous studies provided little indication on the potentiality of meaning extraction from multiple unrecognizable words. In order to see where the limit of this unconscious semantic access lies, reasonably, the next stage is to investigate whether higher-level semantic units can form in the case that meanings need to be extracted and integrated across multiple words.

The few studies in the literature investigating unconscious meaning integration over multiple words have yielded mixed results. On the one hand, a recent continuous flash suppression (CFS) study suggested that meaning integration could occur without consciousness. Using the CFS technique to render the stimuli invisible, Sklar et al. [14] showed that simultaneously presented multi-word expressions could be unconsciously processed for meaning, as three-word sentences that contained semantic violations broke inter-ocular suppression more quickly than those that did not contain semantic violations (e.g., “I drank clothes” vs. “I drank water”). These results are consistent with other studies that did not particularly focus on meaning integration, including aftereffects induced by apparent motion and biological motion under CFS [15] and implicit learning from crowded items presented sequentially [16]. Together, these studies suggested that consciousness may not be necessary for meaning integration from multiple items (termed the *unconscious-integration account* hereafter).

However, on the other hand, a recent study by van Gaal et al. [17] suggested that consciousness is requred for meaning integration, at least for integration among multiple temporally separated words (termed the *conscious-integration account* hereafter). van Gaal and colleagues showed that multiple masked words could be integrated into an unconscious negation so as to produce a priming effect on the following visible target. However, the integration only occurred when the consciously unrecognizable words were presented simultaneously in the same frame (as in their Experiment 2), rather than when words were sequentially presented across different frames (their Experiment 1). These findings are also consistent with recent theories of consciousness that emphasized the role of consciousness in information integration and that information integration is less likely to occur without consciousness [18,19,20].

Extant data thus suggest that temporal segregation between words is a crucial factor for investigating multi-word meaning integration without consciousness. Although unconscious meaning integration may occur when multi-word expressions were simultaneously presented (as demonstrated with the mask technique in [17] and the CFS technique in [14]), unconscious integration for temporally segregated words has not been demonstrated to date. The only study that has investigated unconscious meaning integration over multiple sequentially presented words used the masking paradigm [17] and has found no evidence for integration. Under the backward masking paradigm, however, words in the context were presented only very briefly (for example, 50ms duration in [17]); thus it is possible that there may not have been sufficient time for unconscious temporal integration to occur.

In view of this, we intend to explore here whether multiple consciously unrecognizable words presented sequentially over a longer duration can be bound together to form a holistic phrase-level meaning. This question about how far semantic processing can go in the absence of awareness is not only important for understanding the extent and mechanism of semantic processing in visual crowding, but is also helpful for clarifying the functions of consciousness. In particular, the current study aims to help reveal what exactly is blocked under visual crowding: the semantic processing above a certain level or only the access to consciousness. Furthermore, it could provide evidence for the kinds of integration that can occur without awareness of the stimuli.

To that end, we used the crowding technique to render processes unconscious. The visual crowding technique can present individual words for much longer presentation times than does masking before words are consciously recognized. Furthermore, visual crowding also presents information in a fashion similar to natural reading, in that individual words are almost always presented and read with closely surrounding words instead of in isolation. In addition, we presented Chinese idioms under visual crowding one word at a time. We chose Chinese four-character idioms as our testing materials for their fixed collocation, familiarity, and their implicated holistic meanings. Four-character idiom (or /chengyu/) is a type of traditional Chinese idiomatic expressions, which consists of four Chinese characters. Nearly all characters in four-character idioms we chose for this study are single-character words, and even the non-word characters also each have word-like meaning which is unspecific until put in a certain semantic context. Therefore, we will use “four-word idioms” hereafter to better illustrate our purpose of testing temporal integration of multiple words. Since these four-word idioms are fixed expressions and are highly familiar to Taiwanese students, alterations in any of the component words can be easily detected as incorrect. Moreover, nearly every Chinese four-word idiom has a background story or is related to a historical event, and thus each of the idioms carries abundant information, which can form a strong semantic context. Indeed, many studies have demonstrated N400 mismatch effects using incongruent endings of Chinese four-word idioms, which are not the usual word formation of the idiom but are often syntactically correct (for example [21, 22]). Given all these features, the four-word idioms are ideal for examining semantic integration with a priming paradigm.

Thus, in the present study, Chinese four-word idioms were presented sequentially with either the original congruent ending or a semantically incongruent one. Behavioral (Experiment 1) and Event-Related Potential (ERP) responses (Experiment 2 and 3) to the idiom-final words were assessed. If the three preceding crowded words of the idiom are successfully integrated so as to provide a semantic context for the ending word, we would expect to see a congruency effect between the congruent and incongruent endings, with faster reaction times or less negative N400s in the congruent than in the incongruent condition. However, if the information cannot be successfully integrated over an idiom, no response differences should be observed between the congruent and incongruent conditions. To ensure that our findings are not just confined to idioms, we used the shortest form of temporal integration, two-character words, in the final experiment to see whether the same results can also be obtained as for idioms.

## Experiment 1

To investigate whether meanings of individual words from an idiom can be integrated into a holistic meaning under visual crowding, we first examined the reaction times to idiom-final words that either matched or mismatched the preceding context in meaning. Four-word idioms were presented word by word, with the first three words presented either with flankers (the crowded condition) or without flankers (the isolated condition) and all the final words presented without flankers. The isolated condition serves as a baseline condition for measuring temporal integration over multiple words. Half of the idioms ended with a word and the other half ended with a non-word. Among the real word endings, half were original endings of the idioms (semantically congruent) and the other half were endings incongruent in meaning. Participants were instructed to perform a lexical decision task (LDT) on the last words. This orthogonal manipulation of trial sequence (idiom) and task (LDT) has an advantage that it provides an indirect measure of responses, and because the task is irrelevant to the idiom (or more precisely, the three leading words of the idiom), it can prevent participants from adopting any particular strategies (e.g., predicting the ending word based on the context).

If sequentially presented words can be successfully integrated under visual crowding (the *unconscious-integration account*), we would expect to observe a congruency effect on the idiom-ending words, i.e., faster responses for congruent ending words than incongruent endings words, in both crowded and isolated conditions. In contrast, if meaning integration cannot occur under visual crowding (the *conscious-integration account*), we would predict a reliable congruency effect in the isolated condition only.

### Methods

#### Design

A 2 (Crowding: isolated, crowded) × 2 (Ending: congruent, incongruent) × 2 (Lexicality: word, non-word) within-subject design was adopted, rendering 8 conditions. Each condition contained 20 trials, resulting in 160 trials in total.

#### Participants

Thirty college or graduate students were recruited from the National Taiwan University and were paid with cash for their participation. Participants had normal or corrected-to-normal vision and no history of neurological/psychiatric disorders or brain damage. All gave written informed consent. The study has been approved by the Research Ethics Committee at the National Taiwan University.

#### Apparatus and stimuli

Participants performed the experimental task individually in a dimly lit room, and were seated 60 cm from a computer screen. All stimuli were presented with a black background on a ViewSonic 22” CRT monitor with a spatial resolution of 1,024 × 768 pixels at a 60 Hz refresh rate.

One hundred and sixty commonly-used Chinese four-word idioms were selected for this experiment. For each participant, the computer randomly picked out 80 idioms from the idiom list and assigned them to the crowded condition; the remaining 80 idioms were thus assigned to the isolated condition. In each of the conditions, half of the idioms were presented with incongruent endings. The incongruent- and corresponding congruent-ending words have identical number of strokes and were matched for word frequency (congruent vs. incongruent: 1595 vs. 1574, *t*(159) = 0.15, *p* > .05). The incongruent-ending words have no obvious semantic relation to the three leading words, and cannot be easily integrated with the leading words to form a coherent meaning. In addition, congruent and incongruent ending words shared no pronunciation similarity for both consonants and vowels so as to avoid confounding effects induced by phonological associations.

To render visual crowding, seven additional non-words were created as flankers, from which four flankers were chosen randomly for each crowded word. Each flanker and word occupied 2° × 2° in visual angle. All words were presented in the upper visual field (5° above the fixation). Crowded words were presented with flankers on left, right, top, and bottom of the word, with a center-to-center distance of 2° between the word and each of the flanker (i.e. zero space between the outer edge of the word and flankers).

#### Procedure

**Fig 1:**
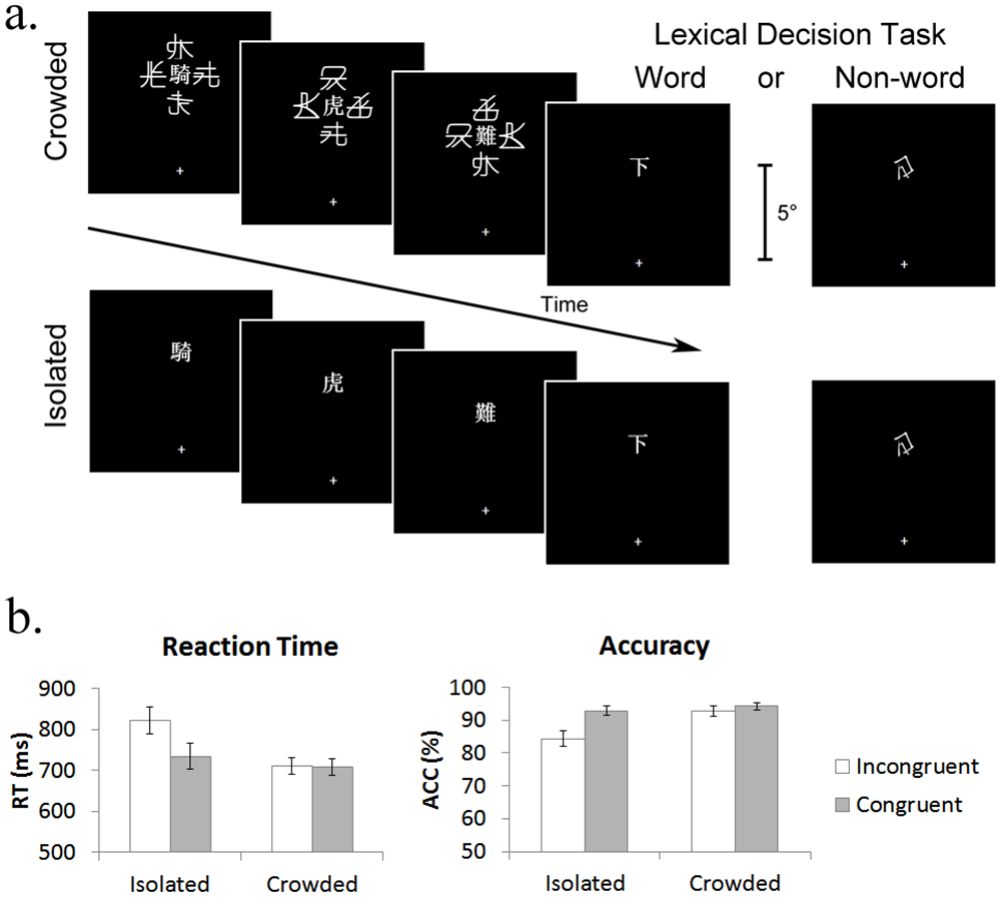
The procedure and results of Experiment 1. (a) Each word of the idiom was presented sequentially at the upper visual field in the crowded or isolated condition. In half of the trials (word condition), the ending word was either congruent or incongruent with respect to the three leading words; in the remaining trials (non-word condition), the last word of the idiom was replaced by a non-word. The idiom illustrated here is “騎虎難下” in the word-congruent condition. This idiom means “Riding a tiger is hard to get off”, referring to a scenario when someone is committed to a difficult task that he/she finds impossible to back out of. In the word-incongruent condition, the last word was replaced by “上” (get on), resulting in an incongruent message: “Riding a tiger is hard to get on”. The participants were asked to perform the LDT to the last (target) word. The mean RT and accuracy of the task were shown in (b). Error bar represents one standard error of the mean.

The procedure is illustrated in Fig 1a. Each trial began with a central fixation (500ms), and then the first three words of an idiom (each presented for 500ms, as in our prior study [12], with a 50ms blank screen in between), followed by an uncrowded ending word that stayed on the screen until response or 2s after the onset. In the crowded condition, words were closely surrounded by four flankers, which rendered the targets consciously unrecognizable. In the isolated condition, words were presented alone without the flankers and were thus consciously recognizable. The participants were required to judge whether the ending word was a word or not with a button-press response. After response, the words were removed and the next trial began after a 1000ms inter-trial interval.

#### Eye movement monitoring

To ensure that participants were fixating at the center of the screen all the time such that the peripheral presentation of crowded words was valid, participants’ eye movements were monitored by an EYELINK 2000 eye tracker (SR Research, Mississauga, Ontario, Canada) with a sampling rate of 1000 Hz. Throughout the experiment, if the eye tracker detected a gaze beyond an invisible line 1.5° above the fixation cross, the experiment would be suspended with all stimuli except the fixation removed until the gaze returned to the fixation cross. The participants were well informed about this before the experiment.

### Results

The overall accuracy of the LDT was 91.2%, and the accuracy in each condition was presented in Fig 1b. The accuracy data were submitted to a 2 (Crowding: isolated, crowded) × 2 (Ending: congruent, incongruent) repeated-measures ANOVA. The results revealed significant main effects of Crowding (*F*(1,29) = 13.12, *p* < .01, *η*_*p*_^2^ = .31) and Ending (*F*(1,29) = 11.30, *p* < .01, *η*_*p*_^2^ = .28), as well as their interaction (*F*(1,29) = 8.48, *p* < .01, *η*_*p*_^2^ = .23). These effects were driven by the performance decrease for the incongruent ending in the isolated condition. LSD post-hoc tests showed that accuracy was significantly impaired for incongruent ending compared to congruent ending in the isolated condition (84.3% vs. 92.8%, *p* < .001), but not in the crowded condition (92.7% vs. 94.2%, *p* = .79).

The ANOVA on the RT data of correct trials also indicated significant main effects of Crowding (*F*(1,29) = 17.33, *p* < .001, *η*_*p*_^2^ = .37) and Ending (*F*(1,29) = 14.13, *p* < .01, *η*_*p*_^2^ = .33), and their interaction (*F*(1,29) = 7.90, *p* < .01, *η*_*p*_^2^ = .21). Consistent with the accuracy results, the responding speed was impaired for incongruent endings in the isolated condition (incongruent vs. congruent: 822ms vs. 734ms, *p* < .001), but not in the crowded condition (711ms vs. 709ms, *p* = .57).

Taken together, the accuracy and RT results suggest that congruency effect occurred only in the isolated condition. The consistent results in both accuracy and RT data indicate no speed-accuracy trade off. To further quantify the evidence against the null hypothesis of no effect for the crowded condition, we performed a Bayesian t-test [23,24,25] for the difference between congruent and incongruent conditions, on both the accuracy and RT data. The Bayes factor (*BF*_10_) for the crowded condition is 0.20 for accuracy (which means that the null effect hypothesis is 4 times more likely to be true than the alternative hypothesis) and 0.32 for RT. That both Bayes factors for accuracy and RT are lower than 1/3 suggested a strong evidence for *H*_0_ [26]. Thus, the results strongly support the hypothesis that no congruency effect occurs under visual crowding.

To ensure that the congruency effect in the isolated condition was indeed due to semantic integration instead of sequence familiarity, and to examine possible semantic integration effects under visual crowding with an on-line measure instead of downstream measures (accuracy and RTs), we assessed participants’ ERP responses in the next experiment. In particular, we focused on the N400 response, a well-characterized component that has been associated with semantic processing and integration [27,28] and shown to reflect semantic integration in unconscious conditions [17,29,30,31,32].

## Experiment 2

Similar to Experiment 1, idioms were presented word by word with the very last word either match or mismatch with the preceding words in meaning. If the meaning of individual words could be accessed and integrated successfully, then the incongruent endings should lead to more negative N400 responses in comparison to the congruent endings. Different from Experiment 1, however, all four words in an idiom were presented with flankers either in spatial proximity with the target (the crowded condition) or distant from the target (the uncrowded condition) to reduce visual differences between conditions. In the uncrowded condition, targets were consciously recognizable, as flankers were distant from the targets.

To avoid interferences from overt movements on the ERP responses of interest, participants were not required to make any response to the last words of idioms. However, to engage the participants during the experiment and to encourage them to attend to the idioms, each idiom was followed by a probe and participants were required to perform a working memory task by judging whether the probe appeared in the preceding idiom or not. This memory task also served as a manipulation check to ensure that the crowded words were indeed unrecognizable to the participants.

### Methods

#### Design

A 2 (Crowding: uncrowded, crowded) × 2 (Ending: congruent, incongruent) within-subject design was adopted, rendering four conditions. Each condition contained 40 trials, resulting in 160 trials in total.

#### Participants

Another group of 31 students participated in this experiment. One participant was excluded from further analysis (see the Results section for details). All participants had normal or corrected-to-normal vision, and had no history of neurological/psychiatric disorders or brain damage. All gave written informed consent.

#### Apparatus and stimuli

Participants were seated about 76 cm from a computer screen in a dimly lit room. All stimuli were presented with a black background on an AUSS 22” LED monitor with a spatial resolution of 1,920 × 1,080 pixels at a 60 Hz refresh rate. The set of idioms used here were identical to those in Experiment 1.

#### Procedure

**Fig 2:**
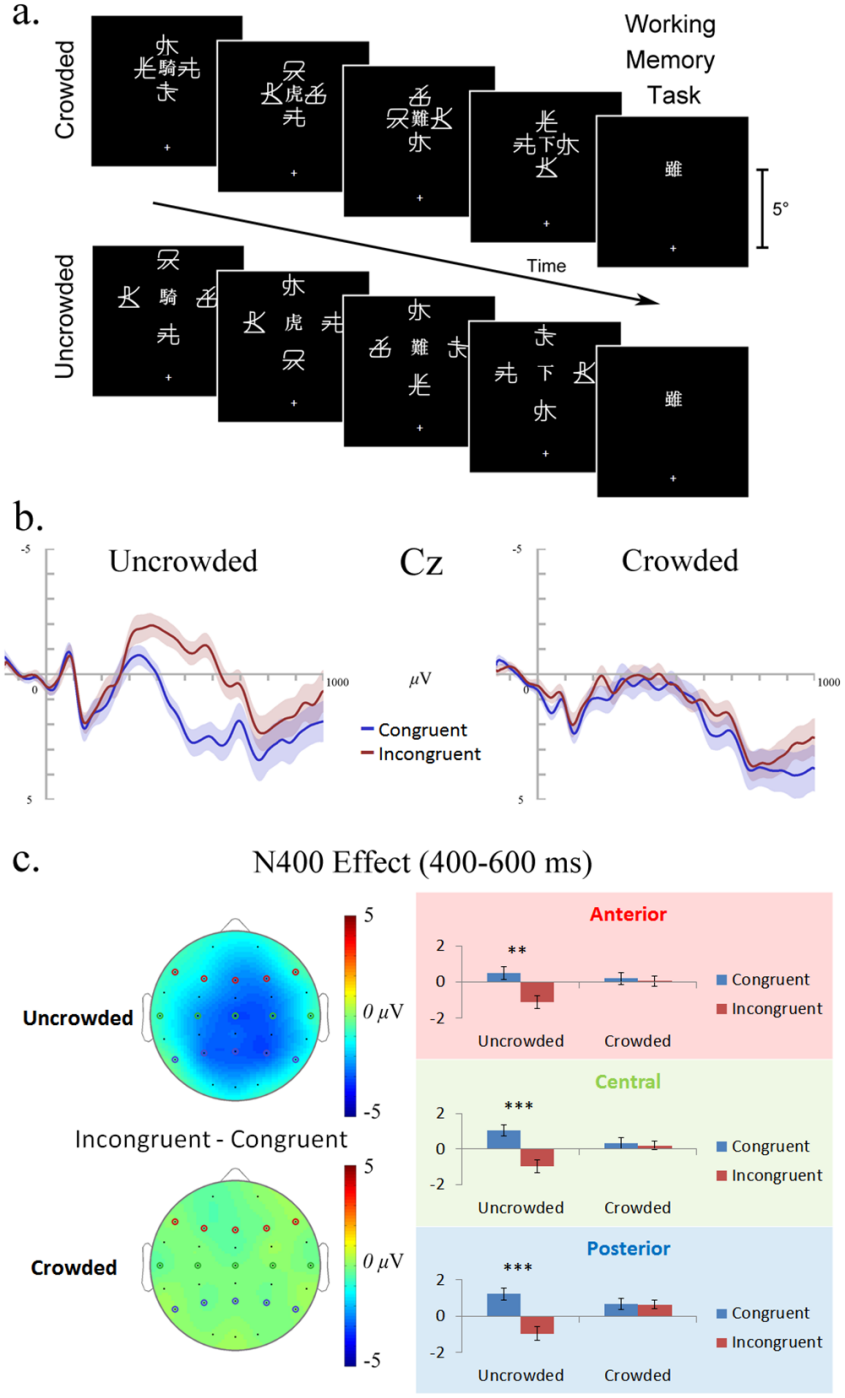
The procedure and results of Experiment 2. (a) Idioms were presented word by word in the crowded and uncrowded conditions, with a fixation presented at the centre of the screen. In each condition, half of the trials contained idioms with a congruent ending word, and the other half contained idioms with an incongruent ending word. Following the idiom, a probe word was presented, which was identical to a word that had just appeared or was a new word that had not appeared in the trial. Participants were required to judge whether the probe word was in the idiom just presented (i.e., the working memory task). Here shows the congruent condition the same idiom as in Fig 1a, and a probe word that had not appeared in the idiom. (b) ERPs for the incongruent (red) and congruent (blue) endings at the representative (for N400) electrode Cz in the uncrowded and crowded conditions. The shaded regions of matching color indicate the ±1 SE between participants. (c) The N400 effect (incongruent minus congruent endings, 400~600ms) in the anterior, central, and posterior regions (averaged across representative electrodes, anterior: F7, F3, Fz, F4, F8; central: T7, C3, Cz, C4, T8; posterior: P7, P3, Pz, P4, P8) in the uncrowded and crowded conditions. The symbols (* p < 0.05, ** p < 0.01, *** p < 0.001) indicate the significance level of the difference between the congruent and incongruent conditions. The topographic maps of the N400 effect are shown in the left panel.

Each trial began with a central fixation (1000ms), then an idiom was presented word by word; each word was presented for 500ms with a 50ms ISI. All stimuli were presented in the upper visual field (5° above the fixation). In the crowded condition, words were closely surrounded by four flankers and were thus consciously unrecognizable. In the uncrowded condition, the spacing between words and flankers was increased to 2°, so that the words could be consciously recognized. Same as in Experiment 1, the ending words of the idioms were either congruent or incongruent. Following the idiom, with an inter-stimuli interval (ISI) of 500ms, a probe word was presented at the same location, in the same font and size. The probe word could be an old word that had appeared in the preceding idiom (i.e., a word picked randomly from the idiom); or a new word that had not appeared in the presented idiom (selected randomly from the other idioms). New and old probes each appeared 50% of the time. Participants were required, in a working memory task, to report whether the probe word had appeared in the sequence, by pressing keys on a standard keyboard. Participants were encouraged to remember the idioms and to guess the answer if individual words could not be clearly identified. This working memory task was conducted to ensure that the participants paid attention to the presentation of the words and to check the subjective visibility of the words, while the research interest was on the ending words (congruent vs. incongruent ending) of the idioms, as indexed by the N400 component. After response, participants were given an opportunity to take a break or blink and were told to press the space bar for the next trial when they were ready. As in Experiment 1, the same gaze monitoring procedure was adopted. Fig 2a illustrates the procedure.

#### EEG acquisition

EEG was recorded from 32 channels using the Neuroscan system. All recordings were initially referenced to a vertex reference electrode, and re-referenced offline to the average of the left and right mastoids. Vertical electrooculogram (vEOG) and horizontal electrooculogram (hEOG) were recorded with two pairs of electrodes: one pair placed above and below the left eye, and the other pair placed at the external ocular canthi of both eyes. Electrode impedances were kept below 5 KΩ. EEG and EOG signals were amplified by the SynAmps using a 0.05–100 Hz bandpass filter and continuously sampled at 1000 Hz/channel for off-line analysis.

EEG responses time-locked to the onset of the last (fourth) words of the idioms were analyzed with the Neuroscan SCAN Edit software. Epochs of EEG data were taken from 150ms before stimulus onset to one second after. EEGs were corrected for ocular artifacts before additional artifact rejection was applied to all scalp electrode sites when trials with EEG amplitudes exceeding ±75μV were removed. On average, 14.2 ± 3.8% of trials were excluded from further ERP analysis. Artifact-free ERPs were then averaged by stimulus type after subtraction of the 150ms pre-stimulus baseline. Prior to statistical analyses, ERPs were digitally filtered with a bandpass filter of 0.05–30 Hz.

### Results

#### Behavior

To ensure that words in the crowded condition were indeed unrecognizable, participants with accuracy in the memory task higher than 75% in the crowded condition were excluded from further analysis. One participant was thus excluded because of the overly high accuracy (96% for crowded trials). For the rest of the 30 participants, the mean accuracy of crowded trials was 60%, and that of uncrowded trials was 87%. The difference in accuracy between the crowded and uncrowded conditions was statistically significant (*t*(29) = 13.66, *p* < .001), indicating that the words in the uncrowded condition were more recognizable than those in the crowded condition.

#### ERPs

As shown in Fig 2b, an N400 difference was observed in the uncrowded condition, with the incongruent endings eliciting more negative responses than the congruent ones. Such effect, however, did not appear in the crowded condition. To characterize this effect, mean amplitudes of the N400 responses measured between 400ms to 600ms (a typical N400 time window) were submitted to a 2 (Crowding: uncrowded, crowded) × 2 (Ending: congruent, incongruent) × 3 (Anteriority: anterior, central, posterior) repeated-measure ANOVA. Fifteen electrodes from the international 10–20 system were chosen as the representative electrodes, among which F7, F3, Fz, F4, F8 represent the anterior region; T7, C3, Cz, C4, T8 represent the central region; and P7, P3, Pz, P4, P8 represent the posterior region. Greenhouse-Geisser corrections were adopted to correct for violations of sphericity associated with repeated measures when necessary.

Results showed a significant main effect of Ending (*F*(1,29) = 20.44, *p* < .001, *η*_*p*_^2^ = .41), a significant main effect of Anteriority (*F*(2,58) = 6.40, *p* < .01, *η*_*p*_^2^ = .18), and a significant Crowding × Ending interaction (*F*(1,29) = 16.85, *p* < .001, *η*_*p*_^2^ = .37). All the other effects were not significant (*p*s > .12, *η*_*p*_^2^ < .09). LSD post-hoc tests showed that the N400 effect was significant only in the uncrowded condition, in all the anterior (*p* < .01), central (*p* < .001) and posterior (*p* < .001) regions; while in the crowded condition, it was not significant in any of the regions (*p*s > .67). The null effect in the crowded condition is also supported by Bayesian analyses: the Bayes factors (*BF*_10_) for the anterior, central and posterior regions were 0.20, 0.21 and 0.20 respectively, indicating strong evidence for the hypothesis that no N400 effect occurs in the crowded condition.

These results suggest that idioms presented in the uncrowded condition could be temporally integrated and thus the incongruent endings elicited the N400 effect. However, this is not true for the crowded idioms; the absence of the N400 effect indicates no temporal integration from unrecognizable words.

## Experiment 3

In Experiment 1 and 2, each word was presented for 500ms, following the duration used in our prior study [12] which shows successful meaning extraction from individual words. However, it reduces the chance for word meanings to be integrated over multiple words due to difficulty in maintaining previously presented words in working memory. Indeed, temporal integration of information has been found using the visual crowding technique with shorter presentation durations. With a presentation duration time of 153ms and SOA of 247ms, Atas et al. demonstrated that participants were able to differentiate between sequences of three different symbols from reversed orders under visual crowding [16]. Thus, to examine whether unconscious meaning integration can occur with shorter presentation duration, Experiment 3 was conducted with a shortened presentation duration time of 250ms, instead of 500ms used in the two prior experiments. Another group of 29 participants were recruited for this experiment, and all other details were identical to Experiment 2.

### Results

#### Behavior

The mean accuracy of crowded trials was 59%, and that of uncrowded trials was 79%. The difference in accuracy between the crowded and uncrowded conditions was statistically significant (*t*(28) = 8.25, *p* < .001), indicating that the uncrowded words were more recognizable than the crowded words.

#### ERPs

**Fig 3:**
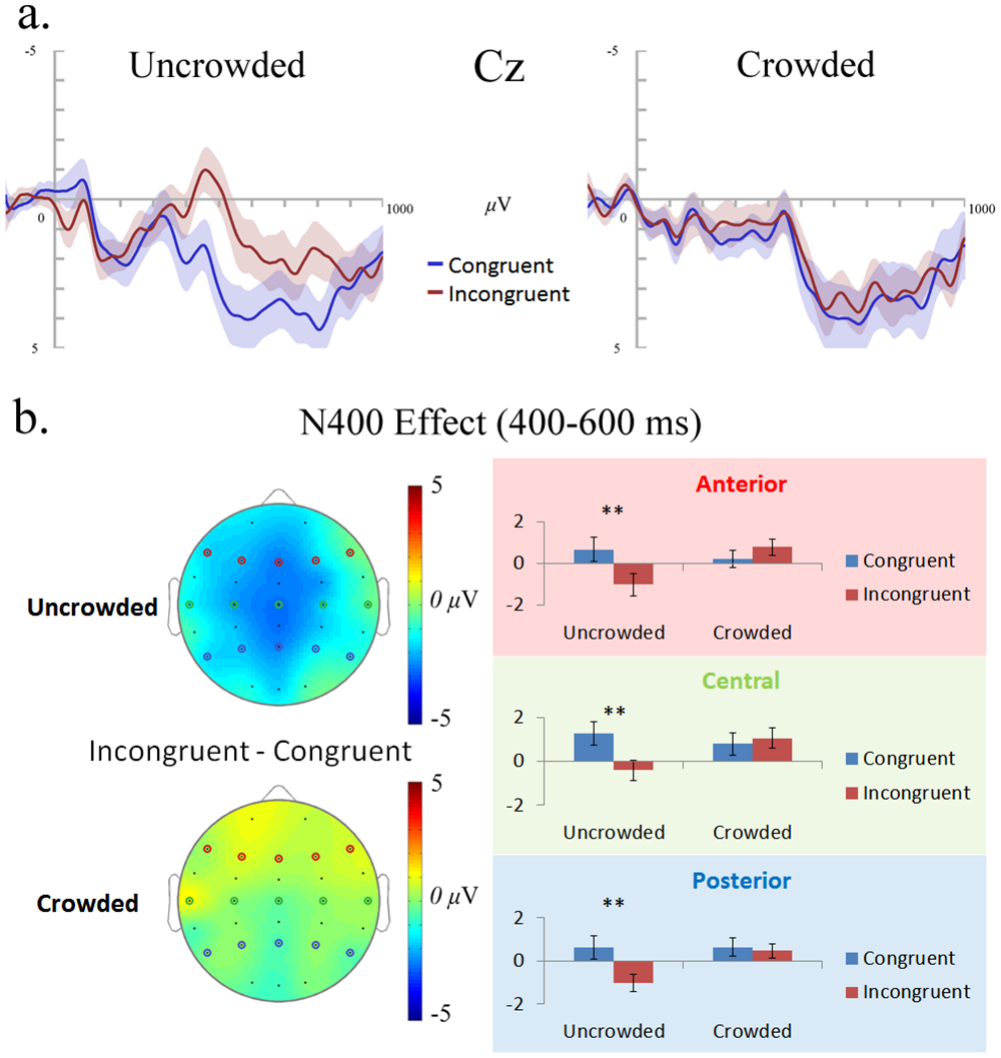
The ERP results of Experiment 3. (a) ERPs for the incongruent (red) and congruent (blue) endings at the representative (for N400) electrode Cz in the uncrowded and crowded conditions. The shaded regions of matching color indicate the ±1 SE between participants. (b) The N400 effect (incongruent minus congruent endings, 400~600ms) in the anterior, central, and posterior regions (averaged across representative electrodes, anterior: F7, F3, Fz, F4, F8; central: T7, C3, Cz, C4, T8; posterior: P7, P3, Pz, P4, P8) in the uncrowded and crowded conditions. The symbols (* p < 0.05, ** p < 0.01) indicate the significance level of the difference between the congruent and incongruent conditions. The topographic maps of the N400 effect are shown in the left panel.

Fig 3 shows the ERP results. ANOVA revealed a marginally significant main effect of Ending (*F*(1,28) = 3.94, *p* = .057, *η*_*p*_^2^ = .12), a significant main effect of Anteriority (*F*(2,56) = 5.04, *p* < .05, *η*_*p*_^2^ = .15), and a significant Crowding × Ending interaction (*F*(1,28) = 9.16, *p* < .01, *η*_*p*_^2^ = .25). All the other effects were not significant (*p*s > .23, *η*_*p*_^2^ < .05). LSD post-hoc tests showed that the N400 effect was significant only in the uncrowded condition, in all the anterior (*p* < .05), central (*p* < .01) and posterior (*p* < .01) regions; while in the crowded condition, it was not significant in any of the regions (*p*s > .29). The null effect in the crowded condition is also supported by Bayesian analyses: the Bayes factors (*BF*_10_) for the anterior, central and posterior regions were 0.33, 0.23 and 0.21 respectively, which is in favor of the null effect hypothesis. Furthermore, in the crowded conditions, the incongruent trials elicited a little more positive amplitude at the N400 time window than that of congruent trials, which is reversed to the effect occurred in the uncrowded condition.

Thus, consistent with the results in Experiment 2, the congruency effect occurred only in the non-crowded condition but not in the crowded condition, even when presentation duration was shortened to facilitate potential temporal integration if it exists.

## Experiment 4

Considering the design of the above experiments, it could be argued that testing four-word idioms before two- or three-word sequences is quite a jump, as the difficulty of integration seems to increase with length (e.g., a reduction of 50% of semantic information for each part of the sequence results in only 6.25% of 4-sequence idiom information). However, such an error accumulation view is based on the assumption that all the words are independent from each other, which is not the case in our study. Chinese four-word idioms are fixed expressions that are well learned by the student participants, so the meaning retrieval of any word in an idiom could affect the meaning retrieval of the rest of the idiom words. Simply put, words in an idiom are not completely independent from each other. Thus, the four-word idioms used as stimuli in the current study are not necessarily harder to integrate than shorter sequences. To highlight this point, in Experiment 4, we used a paradigm similar to that in van Gaal et al.’s study [17] to examine the semantic integration of short word sequence in visual crowding. This time, we presented two-word sequences in visual crowding as primes, which consisted of a modifier (“very” or “not”) and an adjective (“good” or “bad”). Each prime was followed by an isolated noun word target, which was either positive or negative in meaning. Participants were required to report the valence of the target as soon as possible. In this case, if two sequential words (modifier + adjective) were successfully integrated, we could expect a priming effect induced by the two-word negation (“not” + adjective), or only the single-word (i.e. the adjective) priming effect should be observed.

### Methods

Another 20 students participated in this experiment. All participants had normal or corrected-to-normal vision, and had no history of neurological/psychiatric disorders or brain damage. All gave written informed consent.

The prime words included 2 modifiers, “very” and “not”; and 2 adjectives, “good” and “bad”. A set of 32 positive and 32 negative noun words (e.g., “trust” and “tumor”, respectively) were selected as target words. Thus, those stimuli formed 256 different combinations of modifier, adjective and noun words. Each trial presented one of these combinations, resulting in 256 trials in total. All the words were presented in Chinese, and the order of trials was randomized for each participant.

**Fig 4:**
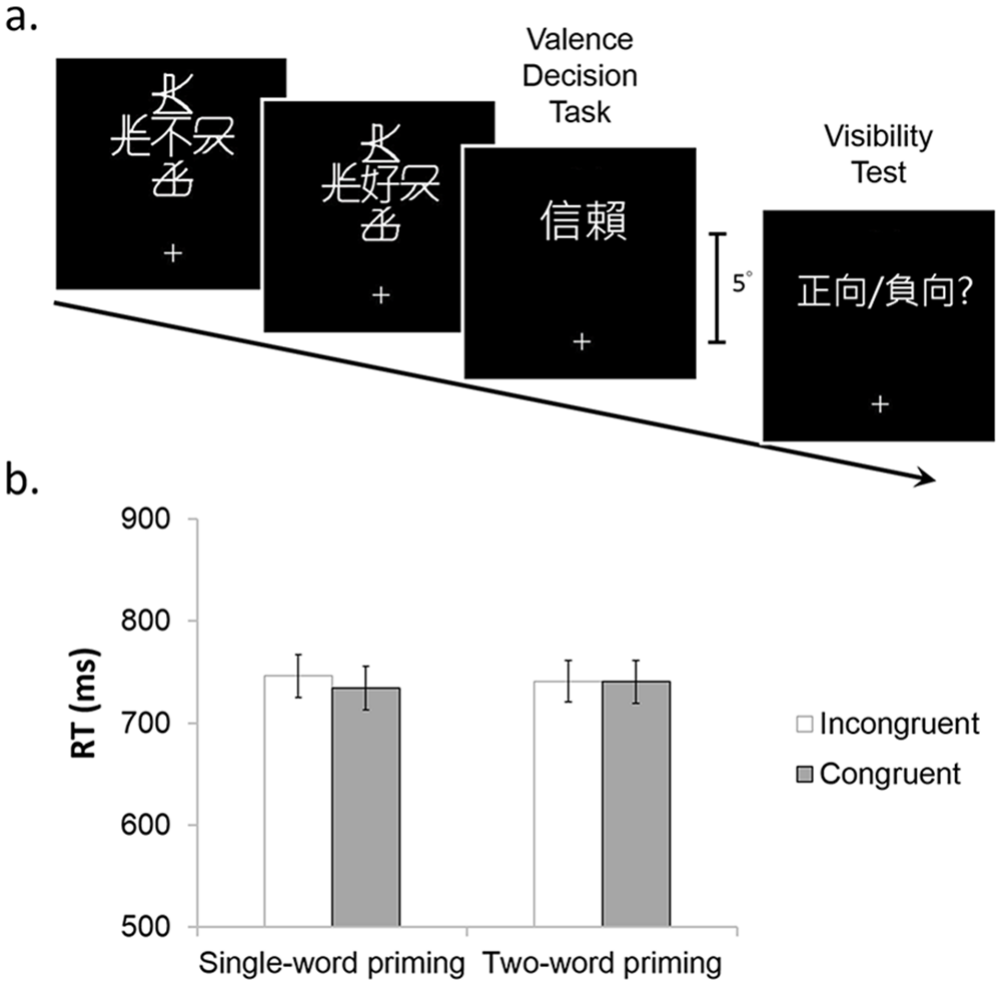
The procedure and results of Experiment 4. (a) A crowded modifier (e.g., “not”) and a crowded adjective (e.g., “good”) were presented sequentially at 5° above the fixation for 250ms, followed by a target word (e.g., “trust”). In half of the trials, the target word would be a positive word, and the target in the other half of the trials would be a negative word. The participants were asked to perform the valence decision task, followed by a visibility test in which the participants had to judge the valance of the two crowded words when combined. The mean reaction time were shown in (b). Error bar represents one standard error of the mean.

Each trial began with a crowded modifier surrounded by four flankers appearing at 5° above the fixation for 250ms. Following that, a crowded adjective was presented at the same location for 250ms as the modifier. After the prime words, a target word appeared at the same location but without flankers. The participant’s task was to make a valence decision—to judge whether the target was a positive or negative word. Visibility tests were administered after each trial, requiring participants to report the valence of the crowded primes (two temporally separated words). Fig 4a illustrates the procedure.

### Results

Single-word and two-word priming effects were analyzed separately. Single-word priming effect was defined as the mean RT difference between adjective-noun incongruent (e.g., bad—trust) and congruent (e.g., good—trust) trials. Because our design of two-word priming considered the effect of negation, congruency was defined as the relationship between noun and the combined meaning of two prime words; the priming effect was measured by the mean RT difference between incongruent (e.g., not good—trust) and congruent (e.g., not bad—trust) trials.

As shown in Fig 4b, paired t-test (one-tail) revealed a significant signal-word priming effect: the response was faster in congruent trials than that in incongruent trials (734ms vs. 746ms, *t*(19) = 2.07, *p* < .05). However, no two-word priming effect was found: the responses were not facilitated in the congruent condition compared to incongruent condition (740ms vs. 741ms, *t*(19) = 0.08, *p* = .47). Thus, even for a short sequence with only two words, temporal semantic integration did not occur in visual crowding.

## General Discussion

Several lines of previous studies have provided empirical evidence for unconscious word integration when multiple words are presented simultaneously [14,17]. However, evidence for unconscious meaning integration over time is still lacking. To our knowledge, the only study that has investigated unconscious multi-word integration over time (and failed to show the integration) used the backward masking technique, which presented words very briefly with a short duration of 50ms and SOA of 117ms [17]. In view of the possibility that the null effect in that study [17] could be due to the overly short presentation duration, in the present study, we used the visual crowding technique to render word processing unconscious for a much longer time (500ms and 250ms in Experiment 2 and 3 respectively) in a manner that is close to real reading when words are presented amidst other words in close spatial proximity.

Even though our crowded stimulus mimicked the perceptual difficulty in real reading, one should note that our temporal idiom presentation was still very different from that. In ordinary reading, idioms are usually processed as a single lexical unit, since the idiomatic meanings can be directly retrieved through their familiar and recognizable configuration [33,34,35]. As well, in Chinese, four-word idiom is linguistically defined as a kind of word, thus treated as a semantic unit. However, this study presented the idioms one word at a time. Such sequential presentation is unfamiliar to participants, therefore invalidating the configurational cues to directly access the idiomatic meanings. In the special case of the current study, there is no other way to retrieve the meaning of the idiom but integrating the sequentially-presented words together.

Chinese four-word idioms were presented word by word for 500ms each under visual crowding. The rationale is that if the first three consciously unrecognizable crowded words in an idiom can be successfully integrated, then they should form a semantic context that is familiar to the participants. With such familiar context, participants should demonstrate differential responses to the congruent versus incongruent endings of the idioms, as they did in the isolated condition. In Experiment 1, with behavioral measure of RT and accuracy from the LDT, showed a reliable congruency effect in the isolated condition: the response to congruent endings was significantly faster than that to incongruent endings. However, no such difference was found in the crowded condition. Both Experiment 2 and 3, using the ERP component N400 as the index, showed a consistent pattern with a reliable N400 congruency effect in the uncrowded condition, but not in the crowded condition. Experiment 4 further addressed the argument that temporal integration of four words was too difficult by verifying the lack of temporal integration with an even shorter sequence comprised of two words. Taken together, the present study adds to the literature on unconscious temporal integration among words ([17], Experiment 1) but provides no evidence of temporally semantic integration under visual crowding, with neither behavioral nor ERP measure. Recalling the work that conflicted with van Gaal et al. [17] and our results, Sklar et al. [14] claimed that simultaneously presented sentence expressions containing semantic violations could break CFS faster than those without semantic violations, which supported the idea of subliminal multi-word integration. However, the major difference between CFS and visual crowding paradigms is that CFS prevents the stimuli from detection, while visual crowding selectively impairs discrimination (or recognizing) of the stimuli [36]. Considering their difference in the functional properties, our results thus suggest that even if detection is preserved, multi-words semantic integration cannot occur in the situation that word discrimination is impaired.

Another important difference between our study and Sklar et al.’s [14] is that we presented words in sequence, while they presented all the words simultaneously under CFS. SSequentially-presented words require temporal integration, which calls for the support of working memory. Working memory is widely thought to be closely related to consciousness ([37,38,39], but see [40] for exceptions). The absence of awareness may block the access to working memory, resulting in failure of temporal integration. Indeed, van Gaal et al. [17] used backward masking paradigm and demonstrated that words presented outside of consciousness simultaneously could be integrated into a negation to trigger the N400 effect on the subsequent target. However, this effect did not occur when the words were presented sequentially with a short duration (50ms) and SOA (117ms). In our study, to ensure successful extraction of meanings from each crowded word, the word presentation duration was initially set to 500ms (Experiment 1 and 2), leading to a total presentation time of about 2 seconds for the idioms, similar to the total duration for the spatial integration of subliminal multi-word integration to occur in Sklar et al. [14]. This also hints at the potential differences between spatial integration shown in Sklar et al. and temporal integration that we explored in this study. At least it can be inferred that temporally-separated presentation is harder for semantic integration to occur without awareness.

To exclude the possibility that the relatively long presentation time (500ms for a word and 2s for an idiom) impedes the semantic integration across time, we conducted Experiment 3 to cut the presentation duration in half to 250ms for each word. Again, we found that the congruency effect occurred only in the non-crowded condition, indicating no semantic integration for crowded words. With a time setting similar to ours (duration time of 153ms and SOA of 247ms), Atas et al. demonstrated evidence of temporal integration over a sequence of three symbols under visual crowding [16], where changes to the sequence order were able to be detected. However, as learning sequential regularities does not necessarily involve the same mechanisms in meaning integration, the current finding suggests such integration cannot occur at the semantic level. Furthermore, the task in Atas et al. was relatively easier than the task used in our current study: as Atas et al. presented the same three-symbol sequence for four times in each trial, any change detection to the relative order of two successive symbols can allow one to differentiate one sequence from the other. In addition, our Experiment 4 demonstrated that sequence length is not the key factor in preventing temporal semantic integration, even after reducing the length to the minimal level—two words, no evidence of successful semantic integration which can lead to priming effect was found.

Finally, we do not exclude the possibility that the measurements in our study lack sensitivity to find the effect induced by unconscious integration. However, since the congruency effect found in the isolated (Experiment 1) and the uncrowded condition (Experiment 2) were quite strong (the incongruent endings slowed reaction by ~100ms in Experiment 1, and induced more N400 responses by ~4 μV in Experiment 2), it seems to suggest that the experimental design as well as the behavioral and ERP measures we adopted were sufficient for generating and capturing stable congruency effects. Nonetheless, we suggest future studies to conduct our experiments utilizing different materials and measures, in order to clarify the boundary of unconscious multi-word integration.

In conclusion, we presented Chinese four-word idioms in visual crowding to examine whether unconscious temporal integration of multiple words would occur. Both behavioral and ERP data suggested that the integration occurred when words were recognizable in the uncrowded condition, but not in the crowded condition. Since our results do not support the existence of unconscious multi-word integration, our findings suggest that integration of temporally separated words might require conscious awareness, at least under the timing conditions tested in the current study.
